# Setting a research agenda for examining early risk for elevated cognitive disengagement syndrome symptoms using data from the ABCD cohort

**DOI:** 10.21203/rs.3.rs-4468007/v1

**Published:** 2024-06-10

**Authors:** Kelsey K. Wiggs, Taryn E. Cook, Isha Lodhawala, Emma N. Cleary, Kimberly Yolton, Stephen P. Becker

**Affiliations:** Cincinnati Children’s Hospital Medical Center; Baylor University; Cincinnati Children’s Hospital Medical Center; Indiana University; Cincinnati Children’s Hospital Medical Center; Cincinnati Children’s Hospital Medical Center

**Keywords:** prenatal risk, perinatal risk, early life risk, cognitive disengagement syndrome, sluggish cognitive tempo

## Abstract

**Background.:**

Little research has examined early life risk for symptoms of cognitive disengagement syndrome (CDS) despite a well-established literature regarding co-occurring outcomes (e.g., attention-deficit/hyperactivity disorder). The current study estimated bivariate associations between early life risk factors and CDS in a large and representative sample of U.S. children.

**Methods.:**

We conducted secondary analyses of baseline data from the Adolescent Brain Cognitive Development (ABCD) study (N = 8,096 children, 9–10 years old). Birthing parents reported early life risk factors on a developmental history questionnaire, including parental, prenatal, delivery and birth, and developmental milestone information. They also completed the Child Behavior Checklist, which includes a CDS subscale that was dichotomized to estimate the odds of elevated CDS symptoms (i.e., *T*-score > 70) in children related to risk indices.

**Results.:**

We observed significantly elevated odds of CDS related to parental risk factors (i.e., unplanned pregnancy, pregnancy awareness after 6 weeks, teenage parenthood), birthing parent illnesses in pregnancy (i.e., severe nausea, proteinuria, pre-eclampsia/toxemia, severe anemia, urinary tract infection), pregnancy complications (i.e., bleeding), prenatal substance exposures (i.e., prescription medication, tobacco, illicit drugs), delivery and birth risk factors (i.e., child blue at delivery, child not breathing, jaundice, incubation after delivery), and late motor and speech milestones in children.

**Conclusions.:**

Several early-life risk factors were associated with elevated odds of CDS at ages 9–10 years; study design prevents the determination of causality. Further investigation is warranted regarding early life origins of CDS with priority given to risk indices that have upstream commonalities (i.e., that restrict fetal growth, nutrients, and oxygen).

## Introduction

Research examining the developmental origins of health and disease hypothesis and/or the prenatal programming hypothesis has demonstrated a wide range of health outcomes in childhood and adulthood are rooted in prenatal and early life experiences [1–4], including neurodevelopmental outcomes (e.g., attention-deficit/hyperactivity disorder [ADHD]) [4]. Although researchers are still pursuing which pre- and perinatal risk factors cause outcomes [5, 6], certain genetic and environmental risk factors are likely to predispose the developing brain to neurodivergence (e.g., ADHD, autism spectrum disorders [ASD]) generally [7–10], while others may specify ultimate developmental outcomes [7, 10].

Cognitive disengagement syndrome (CDS; previously called sluggish cognitive tempo [SCT]) co-occurs in up to 40% of children with ADHD [11–14]. This may suggest etiologic overlap, yet very little is known about the early life origins of CDS [12]. First studied in the 1980s, CDS is characterized by internally-focused distraction (e.g., excessive daydreaming, zoning out), mental confusion, and hypo-activity (e.g., lethargy, drowsiness) [15, 16] .In early factor analytic studies, CDS emerged as a third dimension of ADHD alongside inattentive and hyperactive-impulsive dimensions [15, 16], though it is still debated whether CDS should be considered a presentation of ADHD, its own disorder, or a transdiagnostic phenotypic trait [12, 17, 18]. Furthermore, CDS is related to difficulties (e.g., academic, social, and mental health) beyond what is accounted for by ADHD symptoms alone [12].

Only three studies have examined prenatal risk for CDS [19–21]. Two studies have examined the association between prenatal alcohol exposure and CDS, finding opposing results [19, 20]. Graham et al. [19] found higher mean scores measured by a 15-item CDS scale among children 8–16 exposed prenatally to heavy alcohol use [19]. However, in a more recent study comparing children ages 7.5–9.5 with and without fetal alcohol spectrum disorders, there were no differences between groups regarding the odds of having a CDS score in the “borderline” or “clinical” range (i.e., *T*-score ≥ 65) on the Child Behavior Checklist (CBCL) or Teacher’s Report Form (TRF) [20]. There are many possible reasons for these discrepant findings, including that only 12 children had elevated CDS symptoms in the more recent study [20], which limits statistical power. Finally, Comprodon-Rosanas et al. [21] observed an association between prenatal tobacco exposure and “borderline” or “clinically” elevated CDS scores measured by the CBCL among children ages 7–10, though the association was no longer significant after adjustment for covariates; it should again be noted that only 20 children were observed to have elevated CDS symptoms [21]. In addition to these prenatal studies, there has been one study each documenting an association between extremely low birth weight [22] and iron deficiency [23] in infants with subsequent CDS symptoms in youth.

Identification of early life risk factors for CDS is important because findings regarding common (e.g., to CDS and ADHD) and unique etiologic risk factors may 1) shape ongoing debate and decisions regarding whether CDS is best conceptualized as a distinct phenomenon or as a presentation of ADHD [12], 2) influence prevention and intervention efforts, and 3) guide theory regarding CDS more broadly. The aim of this study was to examine the magnitude of bivariate associations, in a far larger sample than prior literature, to illuminate which risk factors may be most important to prioritize in future investigations.

## Methods

### Data Source

We conducted a secondary analysis of baseline data from the Adolescent Brain Cognitive Development (ABCD) study, which was considered exempt from Institutional Review Board approval. The ABCD study is a prospective, representative twenty-two site study of U.S. children [24]. Baseline data were collected from 2017 to 2018, when children were roughly 9–10 years old. We used data from Release 4.0 in the current study (10.15154/bkya-7b87). Participant recruitment procedures are described elsewhere [25].

### Sample

The ABCD study (N = 11,878) did not include children that were born extremely premature (i.e., born at less than 28 weeks of gestation). For our study, we excluded children who did not have a Developmental History Questionnaire (exposures) completed by their biological mother/birthing parent (n = 1,797), as—like other researchers who have used these data—we anticipated particular difficulty in recall for other caregivers [26, 27]. Next, we excluded children without valid reported birth weight (n = 3), multiples (n = 1,976) [28, 29], and children with missing multiples status (n = 4) [28–31]. Finally, we excluded two children without information on the outcome (see Measures), resulting in a final sample of 8,096 children. We present information on age, sex, and race/ethnicity in Table 1. Of note, from here onward we use the terms birthing parent and other parent to refer to female and male parents, respectively, as we did not have information on gender identity.

## Measures

### Exposures.

We only excluded variables that we believed would be especially susceptible to recall difficulties (e.g., exact ages in months when developmental milestones occurred, dose/frequency of medication and drug use), outside the scope of the current study (e.g., vitamin use), and that were deemed too rare in frequency (i.e., rubella and convulsions in pregnancy). We present the frequencies and percentages of children exposed among the total sample and the elevated CDS group in Table 2. We report on frequency of missing for each variable below.

#### Parental Risk Factors.

Birthing parents reported on whether their pregnancy was planned (missing n = 47, 0.58%), when they became aware they were pregnant (missing n = 514, 6.35%), their age and the age of the other biological parent when their child was born (missing n = 66, 0.82%; missing n = 281, 3.47%; respectively). Because unplanned pregnancies may confer risk to embryonic and fetal development (e.g., via lack of prenatal care in absence of pregnancy awareness) [32] and serve as an indication of broader environmental risk [32], we created an indicator variable for unplanned pregnancy. We also created an indicator variable for pregnancy awareness after 6 weeks of gestation, which is after the observed median in our data (5 weeks) and also after the mean (5.5 weeks) observed in other research [33, 34]. Finally, we dichotomized parental age variables in two ways based on prior literature demonstrating a parabolic pattern of risk for adverse outcomes in children [34–37], including risk for ADHD [38]: teenage (i.e., under 20) and advanced age (i.e., 40 + years for birthing parents and 45 + years for other biological parent).

#### Prenatal Risk: Birthing Parent Illness, Pregnancy Complications, and Substance Exposure.

Birthing parents reported on the presence of the following illnesses and conditions during pregnancy: severe nausea (i.e., beyond 6th month or accompanied by weight loss; missing n = 25, 0.31%), gestational hypertension (missing n = 28, 0.35%), persistent proteinuria (missing n = 40, 0.49%), preeclampsia or toxemia (missing n = 31, 0.38%), gestational diabetes (missing n = 28, 0.35%), severe gall bladder attack (missing n = 15, 0.19%), severe anemia (missing n = 40, 0.49%), urinary tract infection (UTI; missing n = 51, 0.63%), and any other condition requiring medical care (missing n = 15, 0.19%). Pregnancy complications included heavy bleeding requiring bed rest or treatment (missing n = 9, 0.11%), placental problems (e.g., previa, abruptio; missing n = 23, 0.28%), birthing parent accident/injury requiring medical care (missing n = 10, 0.12%), and Rh incompatibility (missing n = 138, 1.71%).

The final set of questions regarding prenatal risk pertained inquired separately about substance exposure in pregnancy (yes/no) prior to and after pregnancy awareness. We created indicator variables for prescription medications, tobacco, alcohol, and illicit drugs (which included prescription medications used to get high and/or not prescribed to them; i.e., oxycontin, benzodiazepines, barbiturates, or amphetamines or methamphetamine, heroine or morphine, marijuana, cocaine or crack, cathinones, fake or synthetic marijuana, GHB, hallucinogens, inhalants, ketamine, MDMA, opioids) throughout pregnancy (i.e., collapsed across pregnancy awareness). For these outcomes, any missing information, including reports by parents that they did not remember, was assumed to mean that the exposure was not present, as we anticipated better recall for these indices. However, it should be noted that that the number of parents who reported not remembering whether substance exposure was present was 497 (6.14%) for prescription medications, 202 (2.50%) for tobacco, 230 (2.84%) for alcohol, and a range of 227 (2.80%; marijuana) to 299 (3.69%; other drugs aside from marijuana, cocaine/crack, morphine/heroin, and OxyContin) for illicit drugs.

#### Delivery, Birth, and Developmental Milestones.

Birthing parents reported on cesarean section (C-section; missing n = 8, 0.10%) delivery, child birth weight (dichotomized for low birth weight ≤ 2500 grams [39]), and whether their child had/experienced any of the following at birth: prematurity (i.e., between 28 weeks and term; missing n = 22, 0.27%), blue at birth (missing n = 80, 0.99%), did not breath at first (missing n = 68, 0.84%), required oxygen (missing n = 70, 0.87%), slow heartbeat (missing n = 81, 1.00%), jaundice that required treatment (missing n = 54, 0.67%), required a blood transfusion (missing n = 15, 0.19%), and incubation after delivery (missing n = 301, 3.72%). Finally, birthing parents rated their perceptions, on a 5-point scale, of whether motor (i.e., sitting, walking, crawling; missing n = 215, 2.67%) and speech milestones (missing n = 215, 2.67%) were met earlier, at the same time, or later than most other children. We created dichotomized variables indicating perceptions of motor and speech milestones being “somewhat later” or “much later” as a marker of broader developmental delays [40].

### Outcome.

Parents completed the Child Behavior Checklist (CBCL), which includes a 4-item (i.e., seems confused or in a fog; daydreams or gets lost in their thoughts; stares blankly; is underactive, slow moving, or lacks energy) CDS subscale within the DSM-oriented scales [41], which are rated on a 3-point scale. Scores on the CBCL CDS subscale correlate strongly with scores on longer, CDS-specific measures [42, 43]. We defined elevated CDS symptoms by a T-score in the clinical range (i.e., ≥ 70); 215 (2.67%) children were categorized as having elevated CDS symptoms (Tables 1 and 2).

## Data Analytic Plan

We calculated odds ratios (ORs) and 95% confidence intervals (CIs) to estimate the magnitude of bivariate associations between exposures and CDS using SPSS version 26 [44]. Given our exploratory aims, each analysis included only those participants who did not have missing information on the variables included.

## Results

**Figure 1:**
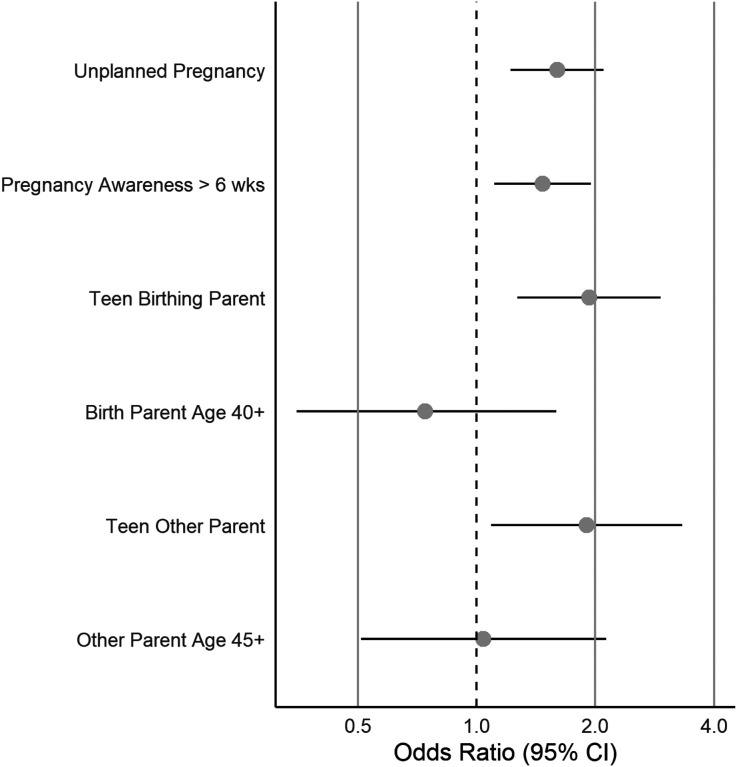
Associations between Parental Risk Factors and Elevated CDS Symptoms

**Figure 2:**
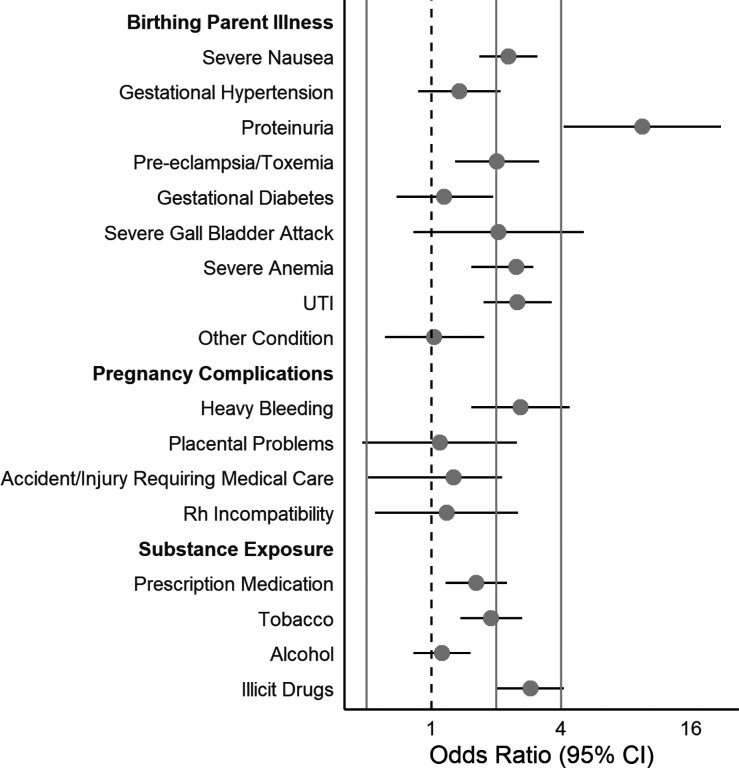
Associations between Prenatal Health Risk Indices and Elevated CDS Symptoms

**Figure 3:**
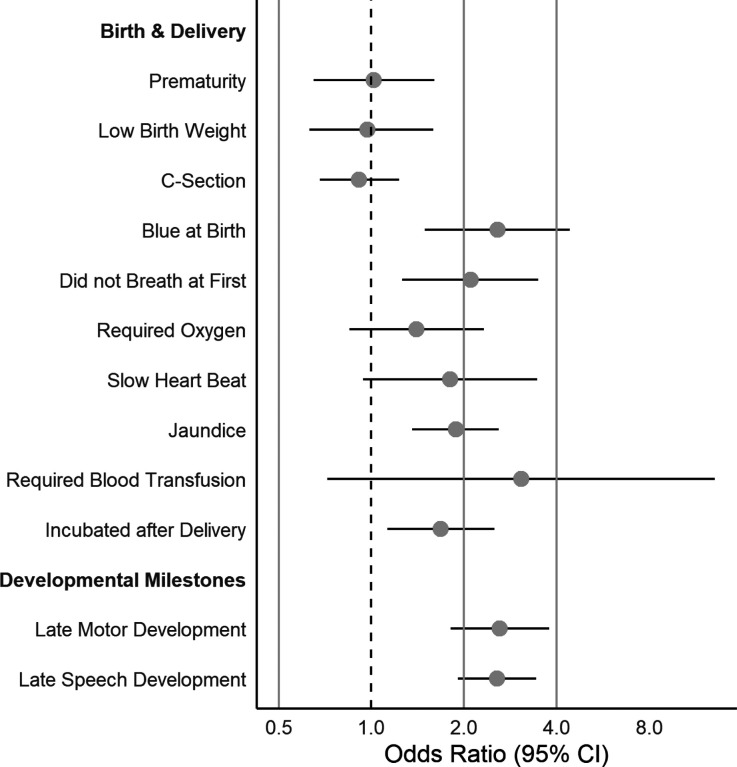
Associations between Delivery, Birth, and Developmental Milestone Risk Indices and Elevated CDS Symptoms

We present ORs and 95% CIs in Table 2 and Figs. 1–3.

### Parental Risk Factors (Fig. 1).

All conception and early pregnancy risk indices were associated with elevated CDS symptoms in children with the exception of advanced age of either parent. We observed 60% higher odds of elevated CDS among unplanned pregnancies compared with planned pregnancies (95% CI = 1.22–2.10), and 47% higher odds among children of parents reporting pregnancy awareness after 6 weeks of gestation relative to earlier pregnancy awareness (95% CI = 1.11–1.95). Teenage parenthood was related to 93% (95% CI = 1.27–2.93) and 90% (95% CI = 1.09–3.32) higher odds compared with birthing and other parents of older ages, respectively.

### Prenatal Risk (Fig. 2).

#### Birthing Parent Illness in Pregnancy.

Of the illnesses parents reported, persistent proteinuria was associated with highest odds of CDS symptoms, with exposed children having a 9.53-fold higher odds than unexposed children (95% CI = 4.12–22.07). Parent-reported preeclampsia/toxemia, was also related over a 2-fold higher odds of clinically elevated CDS symptoms (OR = 2.02, 95% CI = 1.29–3.17). Although parent-reported gestational hypertension was associated with 35% higher odds of CDS, this association was not statistically significant (95% CI = 0.87–2.10). Additional illnesses significantly associated with CDS with over a 2-fold higher odds included severe nausea (OR = 2.28, 95% CI = 1.67–3.12), severe anemia (OR = 2.48, 95% CI = 1.54–2.98), and UTI (OR = 2.51, 95% CI = 1.75–3.62). Associations for gestational diabetes (OR = 1.15, 95% CI = 0.69–1.94) and severe gall bladder problems (OR = 2.05, 95% CI = 0.83–5.11) were elevated but not statistically significant. Finally, we did not observe an association between other conditions requiring medical attention and CDS (OR = 1.03, 95% CI = 0.61–1.76).

#### Pregnancy Complications.

The only pregnancy complication that was significantly associated with elevated CDS symptoms in children was heavy bleeding during pregnancy with 60% higher odds of elevated CDS compared with unexposed children (95% CI = 1.54–4.40). Placental problems, birthing parent accident/injury requiring medical attention, and RH incompatibility were related to slightly higher odds of elevated CDS, but associations were not statistically significant.

#### Prenatal Substance Exposure.

Among prenatal substance exposures, birthing parent-reported illicit drug use was related to the highest odds of elevated CDS symptoms (OR = 2.89, 95% CI = 2.02–4.13). Tobacco exposure (OR = 1.90, 95% CI = 1.37–2.64) and prescription medication use (OR = 1.62, 95% CI = 1.17–2.25) were also related to significantly higher odds of clinically elevated CDS. The smallest magnitude association was observed for alcohol use in pregnancy (OR = 1.12, 95% CI = 0.83–1.52), which was not statistically significant.

### Delivery, Birth, and Developmental Milestones (Fig. 3).

#### Delivery and Birth.

We observed the strongest associations between children being reported as blue at birth and not breathing at first, such that exposed children had roughly a 2.5-fold (OR = 2.57, 95% CI = 1.49–4.41) and 2-fold (OR = 2.10, 95% CI = 1.26–3.49) higher odds of elevated CDS than unexposed children, respectively. Although children requiring oxygen at birth was also related to a 40% higher odds of clinically elevated CDS compared to unexposed children, this association was not statistically significant. Other significant associations were observed for parent-reported jaundice needing treatment (OR = 1.88, 95% CI = 1.36–2.59) and child incubation following delivery (OR = 1.68, 95% CI = 1.13–2.51). Smaller magnitude associations that were not statistically significant included slow heartbeat at birth (OR = 1.80, 95% CI = 0.94–3.46) and children requiring blood transfusions (OR = 3.07, 95% CI = 0.72–13.07). We did not observe elevated risk of CDS related to prematurity (OR = 1.02, 95% CI = 0.65–1.60), low birth weight (OR = 0.97, 95% CI = 0.63–1.59), or C-section delivery (OR = 0.91, 95% CI = 0.68–1.23).

#### Developmental Milestones.

Children of parents reporting late motor and speech development had significantly higher odds of clinically elevated CDS symptoms, both of which were related to over a 2.5-fold higher odds of elevated CDS (motor OR = 2.61, 95% CI = 1.81–3.78; speech OR = 2.56, 95% CI = 1.91–3.43).

## Discussion

Our study aim was to begin establishing a literature examining the prenatal, perinatal, and early life risks for CDS, a construct that co-occurs with other concerns (e.g., ADHD) with more well-established etiological roots in pregnancy and early development. We estimated bivariate associations between a wide range of early life risk factors and subsequent elevations in CDS symptoms in a large and representative sample of U.S. children using birth parent report of their child’s gestational and developmental histories and CDS symptoms at 9–10 years old. Given the dearth of current research in this area, we believe our findings substantially add to the literature and will provide a foundation for research in this area moving forward. Below, we summarize and contextualize our findings with regard for future research needs, beginning with our findings on prenatal substance exposure since this has been the primary focus of prior work. We end with a general discussion of research priorities study limitations.

### Expansion of Current Evidence Regarding Prenatal Substance Exposure and CDS.

Our study is the second that we are aware of to document an association between tobacco exposure in pregnancy and CDS in children. Though Camprodon-Rosanas et al. [21] did not observe a significant association after adjustment for covariates, the study was likely underpowered. However, we did not adjust for covariates given the exploratory aims of the current study, which could also explain differences in findings. In fact, although prenatal tobacco exposure has been robustly associated with fetal growth restriction, prematurity, and small for gestational age [45], which maybe the mechanism by which it influences neurodevelopment [6], there is more mixed evidence regarding whether prenatal tobacco exposure causally contributes to neurodevelopmental outcomes such as ADHD [46–48].

Although parent-reported alcohol use in pregnancy was related to a 12% higher odds of elevated CDS symptoms compared with unexposed children, this is a comparatively small magnitude association, and the estimate was not statistically significant. Thus, our findings are most consistent with Tsang et al. [20], who found no differences in terms of CDS risk, also measured with the CBCL, though using a different cut off. In contrast, Graham et al. [19], who did observe an association, used a more comprehensive, 15-item (rather than 4 items in the CBCL) CDS measure and estimated the association using a total score rather than a binary cut off.

Parent-reported illicit drug use was related to highest risk of CDS among all the prenatal substance exposures examined. Parent-reported prescription medication use was also related to higher risk for elevated CDS symptoms. However, more work is needed to specify which drugs are associated with CDS, as each drug included in the composite variables has distinct pharmacokinetic and pharmacodynamics properties.

### Unplanned Pregnancy, Later Pregnancy Awareness, and Teenage Parenthood May be Proxies for Prenatal Health.

Our findings regarding higher risk of CDS related to unplanned pregnancies, awareness of pregnancy after 6 weeks, and teenage parenthood warrant further investigation. In addition to examination of possible confounding, such investigations should examine the extent to which these variables may be proxies for 1) a lack of prenatal care or 2) exposure to teratogens or other insults in the absence of birthing parent awareness of pregnancy or education regarding prenatal health, which may be the case for teenage pregnant people without support or resources [32, 33]. The prevalence of unplanned pregnancies in the current study is alarmingly high (40.27%), and pregnancy awareness after 6 weeks was prevalent in approximately one fourth (24.77%) of the sample. Although in need of replication and further investigation with regard to CDS specifically, these findings in conjunction with other research [31, 32], highlight the importance of equitable access to sex education, contraceptives, and reproductive and prenatal healthcare.

### Pre- and Perinatal Exposure Indices that Restrict Growth, Nutrients, and Oxygen should be Prioritized in Future Research.

Among birthing parent illness, pregnancy complications, and delivery and birth indices, several patterns emerged providing convergent evidence for higher risk of CDS. First, although parent-reported gestational hypertension was modestly (though not significantly) associated with CDS, we observed the highest risk of elevated CDS symptoms pertaining to persistent proteinuria, with exposed children having almost a 10-fold higher odds of CDS compared to unexposed children. In addition, preeclampsia/toxemia was also significantly related to higher risk of elevated CDS symptoms. Given that gestational hypertension is often a precursor to preeclampsia, the latter of which is also often diagnosed when hypertension is present in combination with proteinuria, these findings provide a consistent narrative regarding the severity and progression of these illnesses in conferring risk for CDS—or alternatively—the importance of considering the confounding factors that may influence and distinguish those who progress from gestational hypertension to more severe conditions from those who do not. It is hypothesized that associations between gestational hypertensive disorders and adverse offspring outcomes may be related to decreased blood flow and oxygen to the placenta [49], which would negatively impact embryonic and fetal growth and access to nutrients and oxygen; these disorders also increase risk of and/or premature labor and delivery [49], which we discuss separately below.

Parent-reported UTI and severe anemia in pregnancy were each associated with approximately 2.5 times higher odds of elevated CDS symptoms compared to children born to parents who did not report these illnesses in pregnancy. These illnesses are also the most common illnesses in pregnancy [50], making further research especially important. Both conditions can also exert effects on placental health and lead to preterm labor [50–52]. Additionally, one study has found iron-deficiency (a common cause of anemia) in infancy to be associated with later CDS in childhood and adolescence [23], which has implications for nutritional deprivation and possible heritable effects of anemia in relation to CDS symptoms [53]. This is also consistent with our finding over double the odds of elevated CDS symptoms related to parent-reported severe nausea and vomiting beyond 6 months of gestation or accompanied by weight loss, which may also impact fetal access to nutrients.

It should be noted that if restriction of fetal growth, nutrients, and/or oxygen were important plausible mechanisms, we might have expected to observe a stronger association for placental problems. Yet, the association was small-magnitude and non-significant. Still, parent-reported heavy bleeding during pregnancy, which is commonly caused by placental problems, was related to higher risk of CDS, and may yet again identify the highest severity placental problems (e.g., significant placental rupture) and confer downstream risk. Furthermore, parent-reported jaundice that required treatment was also related to higher risk for CDS, which can also result from restricted oxygen in utero [54, 55].

Hypoxia or lack of oxygen during labor and delivery may be another important avenue for future research as we observed over double the odds of elevated CDS symptoms related to parent reports of their child being blue and not breathing at first upon delivery compared to children whose parents did not report these exposures. We did not observe statistically significant associations for other related indices, such as slow heart beat at birth and children requiring oxygen after birth, though associations were in the same direction, just smaller in magnitude. Finally, incubation after delivery was also associated with higher risk for CDS, which may be related to many of these risk indices.

Aside from those already mentioned, we observed smaller magnitude and non-significant associations for parent-reported severe gall bladder attack, gestational diabetes, Rh incompatibility, and children requiring a blood transfusion after delivery, which may suggest that these indices should be less of a research priority for future investigations of pre- and perinatal risk for elevated CDS symptoms. We also found no elevations in CDS symptoms when comparing children delivered with and without C-section delivery, premature birth, and low birth weight. We discuss prematurity and low birth weight in more detail next.

### Replication of Prematurity and Birth Weight Findings in a Sample that Includes Extremely Premature Children is Needed.

Our study findings and their potential implications regarding investigation of fetal growth and/or premature labor and delivery are complicated by our lack of finding elevated CDS symptoms related to prematurity and low birth weight. For both of these indices, it is important to highlight that a significant limitation of the current study is the exclusion of children born extremely premature (i.e., prior to 28 weeks of gestation), thus truncating the variance of these variables to a significant degree. Indeed, this may explain why we did not replicate findings of Georgsdottir, Haraldsson [22] who observed an association between extremely low birth weight and CDS symptoms during teenage years. Furthermore, we did not have information on gestational age at birth for children that were born at and beyond term, which also limited our ability to examine indices such as small for gestational age. However, we might expect some of the observed associations in the present study to be strengthened by the inclusion of extremely premature infants, since prematurity and fetal growth restriction are one mechanism by which many of these exposures are hypothesized to take effect. Finally, the prevalence of clinically elevated CDS symptoms in the ABCD cohort is lower than in prior research using nationally representative samples, which may again support the notion that the current sample may not be capturing a subset of children at risk for CDS [11, 13].

### Late Motor and Speech Development may be Markers for CDS.

We observed over a 2.5 times higher odds of elevated CDS symptoms among children whose parents perceived their children to achieve motor and speech milestones later than other children. An important next step for this and for pre- and perinatal health indices will be to rule out the explanation that these associations are only present because of the high co-occurrence between CDS and ADHD (and other neurodevelopmental outcomes).

### Methodological Considerations for Future Research.

In addition to research on the topics discussed above, we want to highlight some important methodological next steps. First, now that we have a better understanding of which early life risk factors most strongly relate to CDS, the next step is to more rigorously examine whether observed associations might be causal. In addition to covariate adjustment, quasi-experimental designs (e.g., sibling comparisons) and methods commonly used in epidemiology (e.g., active comparators, matching) are especially important to rule out confounding by genetic and other environmental factors. Second, the use of more comprehensive measures of CDS (like those used in Graham et al. [19]) will be critical to properly detect associations, as was recently highlighted in a review identifying key directions for CDS research that also called for more etiologic and early life risk factor research [12]. Third, it will be essential to examine common and unique risk factors for CDS and commonly co-occurring difficulties, including other neurodevelopmental concerns (e.g., autism spectrum disorder).

## Conclusions

The current study sought to estimate the magnitude of associations between a broad range of early life risk factors and elevated CDS symptoms in children at 9–10 years of age in a large and representative sample of U.S. children, in order to set a research agenda for future work on this topic. We observed initial evidence that warrants further research in many areas, including prenatal substance exposures, other pregnancy conditions and complications that may restrict fetal growth and access to nutrients and oxygen, and achievement of early life developmental milestones. Future research should prioritize examination of the likelihood that these exposure indices causally contribute to CDS.

## Figures and Tables

**Table 1:** Child Demographics Stratified by CDS Group

	No CDS Elevation (*T*-score > 70; n = 7881)	CDS Elevation (*T*-Score ≥ 70; n = 215)
	Mean (SD)	Mean (SD)
Age at Research Visit	118.51 (7.57) months	118.40 (7.70) months
	n (%)	n (%)
Sex Assigned at Birth		
Female	3786 (48.04)	83 (38.60)
Male	4095 (51.96)	132 (61.39)
Race		
Asian	481 (6.10)	12 (5.58)
Black	1673 (21.23)	67 (31.16)
Native/Indigenous Alaskan or American	266 (3.38)	11 (5.12)
Native Hawaiian or Pacific Islander	44 (0.56)	4 (1.86)
White	5811 (73.73)	144 (66.98)
Other	574 (7.28)	25 (11.63)
Unknown	119 (1.51)	1 (0.47)
Hispanic/Latiné	22 (0.28)	1 (0.47)

Note CDS = cognitive disengagement syndrome (termed ‘sluggish cognitive tempo’ on the Child Behavior Checklist scale used to define the construct in this study).

**Table 2:** Exposure Frequencies Among Total Sample, Elevated CDS Sample, and Odds Ratios Estimating Associations between Exposures and CDS

	Total Sample (N = 8,096)	CDS Elevation (*T*-Score ≥ 70; n = 215)	
	n (%)	n (%)	Odds Ratio (95% CI)
Parental Risk Factors			
Unplanned Pregnancy	3,260 (40.27)	111 (51.63)	1.60 (1.22–2.10)
Pregnancy Awareness after 6 Weeks	2,248 (24.77)	77 (35.81)	1.47 (1.11–1.95)
Teen Birthing Parent	549 (6.78)	26 (12.09)	1.93 (1.27–2.93)
Birthing Parent Age of 40+	348 (4.30)	7 (3.26)	0.74 (0.35–1.59)
Teen Other Parent	292 (3.61)	14 (6.51)	1.90 (1.09–3.32)
Other Parent Age of 45+	290 (3.58)	8 (3.72)	1.04 (0.51–2.13)
Birthing Parent Illness in Pregnancy			
Severe Nausea	1,125 (13.90)	56 (26.05)	2.28 (1.67–3.12)
Gestational Hypertension	678 (8.38)	23 (10.70)	1.35 (0.87–2.10)
Persistent Proteinuria	35 (0.43)	7 (3.26)	9.53 (4.12–22.07)
Preeclampsia/Toxemia	448 (5.53)	22 (10.23)	2.02 (1.29–3.17)
Gestational Diabetes	535 (6.61)	16 (7.44)	1.15 (0.69–1.94)
Severe Gall Bladder Attack	96 (1.19)	5 (2.33)	2.05 (0.83–5.11)
Severe Anemia	338 (4.18)	20 (9.30)	2.48 (1.54–2.98)
Urinary Tract Infection	645 (7.97)	37 (17.21)	2.51 (1.75–3.62)
Any other condition requiring medical care	552 (6.82)	15 (6.98)	1.03 (0.61–1.76)
Pregnancy Complications			
Heavy Bleeding	253 (3.13)	16 (7.44)	2.60 (1.54–4.40)
Placental Problems	209 (2.58)	6 (2.79)	1.10 (0.48–2.50)
Birth Parent Accident/Injury Requiring Medical Care	151 (1.87)	5 (2.33)	1.27 (0.51–2.13)
RH Incompatibility	230 (2.84)	7 (3.26)	1.18 (0.55–2.53)
Prenatal Substance Exposure			
Prescription Medication	1,236 (15.27)	48 (22.33)	1.62 (1.17–2.25)
Tobacco	1,082 (13.37)	48 (22.33)	1.90 (1.37–2.64)
Alcohol	2,043 (25.23)	59 (27.44)	1.12 (0.83–1.52)
Illicit Drugs	601 (7.42)	39 (18.14)	2.89 (2.02–4.13)
Delivery and Birth			
Prematurity	818 (10.10)	22 (10.23)	1.02 (0.65–1.60)
Low Birth Weight	755 (9.33)	20 (9.30)	0.97 (0.63–1.59)
C-section	2,488 (30.73)	62 (28.84)	0.91 (0.68–1.23)
Blue at Birth	241 (2.98)	15 (6.98)	2.57 (1.49–4.41)
Did not Breath at First	335 (4.14)	17 (7.90)	2.10 (1.26–3.49)
Required Oxygen	475 (5.87)	17 (7.90)	1.40 (0.85–2.32)
Slow Heartbeat	218 (2.69)	10 (4.65)	1.80 (0.94–3.46)
Jaundice Requiring Treatment	1,192 (14.72)	51 (23.72)	1.88 (1.36–2.59)
Required Blood Transfusion	26 (0.32)	2 (0.93)	3.07 (0.72–13.07)
Incubated after Delivery	725 (8.96)	29 (13.49)	1.68 (1.13–2.51)
Developmental Milestones			
Late Motor Development	599 (7.40)	36 (16.74)	2.61 (1.81–3.78)
Late Speech Development	1,276 (15.76)	68 (31.63)	2.56 (1.91–3.43)

Note CDS = cognitive disengagement syndrome.

## Data Availability

The DOI for this study through the national data archive can be found at https://nda.nih.gov/general-query.html under the following assignment: DOI: 10.15154/z563-zd24
